# Exome-wide rare variant analyses of two bone mineral density phenotypes: the challenges of analyzing rare genetic variation

**DOI:** 10.1038/s41598-017-18385-9

**Published:** 2018-01-09

**Authors:** Jianping Sun, Karim Oualkacha, Vincenzo Forgetta, Hou-Feng Zheng, J. Brent Richards, Daniel S. Evans, Eric Orwoll, Celia M. T. Greenwood

**Affiliations:** 1 Department of Epidemiology, Biostatistics and Occupational Health, McGill University, Montreal, QC Canada; 2 Lady Davis Institute for Medical Research, Jewish General Hospital, Montreal, QC Canada; 3 Département de mathématiques, Université du Québec à Montréal, Montreal, QC Canada; 4 Institute of Basic Medical Sciences, Westlake Institute for Advanced Study, Westlake University, Hangzhou, Zhejiang, China; 5 Institute of Aging Research and the Affiliated Hospital, School of Medicine, Hangzhou Normal University, Hangzhou, Zhejiang, China; 6 Department of Human Genetics, McGill University, Montreal, QC Canada; 7 California Pacific Medical Center Research Institute, San Francisco, CA USA; 8 Department of Medicine, Bone and Mineral Unit, Oregon Health and Science University, Portland, OR USA; 9 Department of Oncology, McGill University, Montreal, QC Canada

## Abstract

Performance of a recently developed test for association between multivariate phenotypes and sets of genetic variants (MURAT) is demonstrated using measures of bone mineral density (BMD). By combining individual-level whole genome sequenced data from the UK10K study, and imputed genome-wide genetic data on individuals from the Study of Osteoporotic Fractures (SOF) and the Osteoporotic Fractures in Men Study (MrOS), a data set of 8810 individuals was assembled; tests of association were performed between autosomal gene-sets of genetic variants and BMD measured at lumbar spine and femoral neck. Distributions of p-values obtained from analyses of a single BMD phenotype are compared to those from the multivariate tests, across several region definitions and variant weightings. There is evidence of increased power with the multivariate test, although no new loci for BMD were identified. Among 17 genes highlighted either because there were significant *p*-values in region-based association tests or because they were in well-known BMD genes, 4 windows in 2 genes as well as 6 single SNPs in one of these genes showed association at genome-wide significant thresholds with the multivariate phenotype test but not with the single-phenotype test, Sequence Kernel Association Test (SKAT).

## Introduction

The massive advances and cost decreases in sequencing technologies have led to identification of millions of rare minor alleles (defined here as those minor allele frequency (MAF) less than 0.05). However, power to detect associations between complex traits and such genetic variants is low, due to the small number of individuals who carry such variants. Set-based tests have been proposed to try and improve power by jointly testing association with multiple rare variants in a pre-defined set. In set-based analyses, SNPs are assigned to SNP sets by forming linkage disequilibrium (LD) blocks, using sliding windows or on the basis of some meaningful biological criteria (genomic features); e.g., genes or pathways^1^. Many different set-based methods have been proposed, such as burden tests^2,3^, variance component score test^4^, the combination of these two types of tests^5,6^, and many others^7^.

Despite the potential for improved power of set-based methods, statistical power is often limited due to the complex way that genotypes map to the phenotypes, to the complex networking among factors that influence the etiology of diseases^8,9^, and also because picking the genomic set for the test is quite challenging since it is usually unclear which variants should be included. The best way to improve power is, of course, to enlarge sample sizes, and for feasibility reasons, this is often achieved by combining participants from different studies together, either directly or through the use of meta-analysis^10^. Improvements in power can also be achieved through using imputation: ungenotyped variants can often be accurately estimated when the genotyped variants are used as a skeleton framework for imputation against a large panel of individuals who have been sequenced^11^.

Most currently used rare-variant tests analyze one phenotype—that is, they focus on the association between a set of rare variants and a single phenotype. However, multiple correlated phenotypes are often collected. Therefore, there is also potential for increasing power by capturing phenotypic correlations and their relationships to associated genetic variants. Recently, several multivariate rare-variant association tests have been developed^12–14^, and these authors have shown that multivariate tests are generally more powerful than the univariate ones by accounting for existing pleiotropic effects^15^ as well as the correlations among multiple phenotypes.

In this paper, we use a well-studied phenotype, bone mineral density (BMD)^16,17^, to investigate the potential benefits associated with a multivariate-phenotype analysis of correlated BMD phenotypes and associations with genetic variation using set-based methods. To create a data set with reasonable power for rare-variant analyses, a single data set was created by combining individual-level data from three large cohorts: whole-genome sequencing data from a subset of the UK10K project^18^ (http://www.uk10k.org/ and www.gefos.org), and genome-wide genotyping data that had undergone imputation from the Study of Osteoporotic Fractures (SOF)^19^ (http://sof.ucsf.edu/interface/) and Osteoporotic Fractures in Men (MrOS) Study^20,21^. Tests of association between genetic variation in the genetic regions and BMD are performed with a recently developed multivariate rare-variant set-based association test (MURAT)^12^ that is built on a mixed effect model, and the well-known single phenotype Sequence Kernel Association Test (SKAT) test^4^, which is derived similarly from a mixed effect model. We first perform an exome-wide gene-based analysis, and then study more intensively some selected candidate genes.

## Results

### Genome-wide analysis

To assess the impact on power in a genome-wide analysis, we applied region-based tests of association for all genes using SKAT, for the BMD phenotypes at two positions: lumbar spine (LS) and femoral neck (FN), separately, as well as using MURAT for a joint analysis of both phenotypes. Analysis was performed on 19,052 genes, but since some genes were extremely large (greater than 150 kb), we divided the large genes into smaller pieces (called gene pieces), leading to a total of 24,333 regions for analysis (Supplemental Table 1). The number of genetic variants in these gene pieces varied substantially from only 1 up to 2898 variants (Supplemental Figure 1).

**Figure 1:**
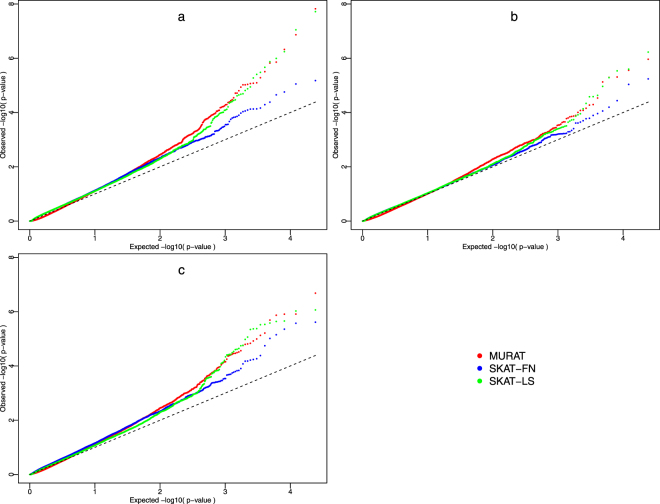
Q-Q plots for genome-wide gene piece analysis with three different variant weights, where the expected quantiles (after -log10 transformation) are shown on the horizontal axes and the -log10 *p*-values on the vertical axis. (**a**) Identical weights. (**b**) Weights based on a Beta (1,25) distribution. (**c**) Weights based on a Beta (0.5, 0.5) distribution. SKAT-FN denotes using SKAT to test the femoral neck BMD; SKAT-LS represents SKAT analysis results for lumbar spine BMD.

Figure 1a shows QQ-plots of *p*-values for these primary results with equal weights applied to all genetic variants. The distributions of *p*-values from univariate and multivariate analyses demonstrate fairly substantial deviations from the line of expectation at the left of Fig. 1, suggesting either that there could be a large number of regions each contributing a small amount to BMD, which is similar to a QTL infinitesimal model^22^, or that there is some population stratification for which we have not fully adjusted. All analyses included, among the covariates, the first 5 principal components derived from the genetic data to account for potential confounding by population substructure. A scree plot of the variance explained by the principal components shows that the first 5 components capture more variability than the remainder (Supplemental Figure 2). Although we recognize that additional components may have improved the QQ-plot fits, our focus here was to compare multivariate and univariate analyses, and we do not feel that the comparisons would be altered by adding additional principal components because both analyses used the same principal components set.

To assess whether the result was sensitive to the weighting of variants within a region, we repeated the genome-wide analysis with two additional weighting schemes. Firstly, variants were weighted following the default choice in SKAT where the weights are assigned based on the probability density of a Beta (1, 25) distribution of the MAF (Fig. 1b), i.e. the square root of the weight for the *j* ^th^ variant equals *beta*(*MAF*_*j*_,1,25)), where *MAF*_*j*_ is the MAF for the *j* ^th^ variant, and *beta*(*x*, *α*, *β*) is the probability density function for a random variable, *x*, coming from beta distribution with parameters α and β. Then a third weight was defined by assuming the MAF follows a Beta (0.5, 0.5) distribution (Fig. 1c), where Beta (0.5, 0.5) is defined similarly to Beta (1, 25). The degree of deviation of the QQ plots from the line of expectation varies with the choice of weighting function, with the largest test statistics appearing in the analysis with identical weights, and the smallest deviations seen with Beta (1,25) weights. It has been previously shown that rare variants are under different selection pressure, and hence may be differentially affected by population structure^23,24^. In fact, larger population differences are expected for rare variants. However, it is also true that power is much better for common variants, and the identical weight analysis gives the most weight to such SNPs. Therefore, the larger deviation seen in the identical weight analysis suggests that the QQ plot deviations may be largely driven by BMD associations rather than population structure.

Near the top of Fig. 1a, the QQ plot for MURAT shows a small increased deviation from the line of null expectation, when compared to the SKAT results, and this suggests potential increases in power for some genes. This finding is attenuated with the other weighting schemes (Fig. 1b and c). It is worth noting that the most significant gene pieces in the analysis with identical weights, at the very top of Fig. 1a, are identified by the multivariate approach with the MURAT test.

To properly interpret the statistical significance of the SKAT results relative to MURAT, the *p*-values from SKAT need to be adjusted for the fact that two phenotypes were analyzed separately. However, the two single-phenotype test results can be expected to be correlated since the two phenotypes in the combined data set are quite correlated (r = 0.64). We performed permutation analysis of the phenotypes versus the genotypes and repeated the region-based analyses on chromosome 9 with different weightings. Using the extrapolation method in Xu *et al*.^25^, we estimated the genome-wide significance threshold at 7.51 × 10^−7^ for MURAT and 1.12 × 10^−7^ for the minimum of the two SKAT tests across all three weights (See Supplemental Text for details). However, the estimated SKAT threshold is, in fact, smaller than a Bonferroni threshold (0.05/(3 weights by 2 phenotypes by 24,333 genes) = 3.42 × 10^−7^. Since we would expect an estimated threshold that takes correlation into account to be *less* stringent than Bonferroni, we propose to use the Bonferroni threshold to establish significance for SKAT.

Therefore, using these thresholds, we identified three gene pieces as showing significance with MURAT and two genes with SKAT. We assumed these thresholds would apply, approximatively, for the other weighting schemes, and therefore Table 1 shows all gene pieces having significant associations with this genome-wide region-based analysis. All these identified genes were analyzed in their entirety as a single region (i.e. none of these genes needed to be separated into gene pieces); also, all these genes have been previously reported by Zheng *et al*.^16^ as associated with BMD.

**Table 1:** Significant gene pieces identified by MURAT or SKAT under three different choices of weights. The significance thresholds used are 7.51 × 10^−7^ for MURAT and 3.42 × 10^−7^ for SKAT.

Chr	Gene	Start Position	End Position	#SNP	Percent of rare variants	*p*-value	
						adj.SKAT	MURAT
*Identical Weight*							
1	WLS	68559219	68703602	939	59.5%	8.91 × 10^−8^	4.74 × 10^−7^
8	TNFRSF11B	119930833	119969157	375	68.5%	5.72 × 10^−7^	1.36 × 10^−7^
8	COLEC10	120002915	120123590	852	67.0%	1.92 × 10^−8^	1.51 × 10^−8^
*Beta(0.5,0.5) Weight*							
8	TNFRSF11B	119930833	119969157	375	68.5%	2.66 × 10^−6^	2.07 × 10^−7^

The adjusted *p*-value for SKAT is defined as the minimum of two *p*-values obtained by testing LS and FN individually.

From Table 1, we can see that SKAT only identified two significant genes under identical weight, both of which were also significant under MURAT with identical weight, and no genes are significant under the Beta (1,25) weight.

For comparison, we also applied Maity’s approach^14^ with identical weights to the three significant genes listed in Table 1, however, no evidence of significant association was found with that method. After further investigation, we believe the reason for this result is that MURAT also considers possible correlation between variant effects, unlike Maity’s method, which only considers the correlation among multiple traits. Consequently, MURAT has the ability to capture pleiotropic effects and thereby improve power. In fact, Maity’s approach is a special case of MURAT when the assumed common correlation between variant effects, *ρ*, is zero. Table 2 shows that if we apply MURAT with identical weights but with *ρ* = 0 to these three genes, the resulting MURAT *p*-values are similar to those obtained from Maity’s method. However, since in MURAT we make a grid search across *ρ* from 0 to 1, we can see that MURAT obtains much smaller *p*-values at other values for *ρ*, and finds a non-zero optimal *ρ* (Table 2).

**Table 2:** The *p*-values for three significant genes listed in Table 1 when Maity’s approach with identical weight is applied.

Chr	Gene	Maity	MURAT	
			ρ = 0	Optimal ρ
1	WLS	1.33 × 10^−2^	1.39 × 10^−2^	2.50 × 10^−7^
8	TNFRSF11B	1.30 × 10^−3^	8.87 × 10^−4^	7.80 × 10^−8^
8	COLEC10	1.50 × 10^−3^	1.39 × 10^−3^	6.64 × 10^−9^

The last two columns are *p*-values obtained via MURAT with identical weight at *ρ* = 0 and at the optimal *ρ*, where optimal *ρ* is defined as the one that gives the smallest *p*-value within a grid of *ρ* from 0 to 1 by 0.1. The optimal *ρ*’s for these genes are 0.9, 0.7, and 0.7, respectively.

This region-based analysis of gene pieces did not identify many of the genes well-known to contain variants influencing bone mineral density^17^. This is a common consequence of set-based analyses that group together a probably-small number of associated variants with many variants of no effect, and such attenuations of power have also been examined through simulation studies in Li and Leal^2^ and Ladouceur *et al*.^26^. Therefore, in Supplemental Table 2, we also report our *p*-values at 14 other well-replicated bone-density-associated genes, and for some of these genes, suggestive *p*-values (*p*-values < 0.01) are seen at RUNX2, ESR1, WNT16, DKK1, SOX6, LRP5, SP7, TNFSF11 and SOST for at least one gene piece and weighting scheme. Certainly, however, their *p*-values would not stand out in a genome-wide analysis.

Nevertheless, our gene pieces contain as many as 2800 variants, and the effects of a small number of causal variants could be overwhelmed in such regions. Therefore, we undertook a second set of analyses of candidate genes using smaller windows containing a maximum of 30 variants, to further compare multivariate and univariate region-based test performance.

### Small window sensitivity analyses

To investigate MURAT’s performance in more depth at known BMD genes, we selected 17 genes for analysis with smaller non-overlapping windows containing no more than 30 variants. This list contains the 3 genes in Table 1, as well as 14 additional genes with known contributions to BMD (Supplemental Table 3). In total, there were 659 small genomic segments included in this sensitivity analysis.

If we retain 3.42 × 10^−7^ and 7.51 × 10^−7^ as the SKAT and MURAT thresholds for statistical significance (see Supplemental Text), there are 21 and 7 variant segments detected by either SKAT or MURAT under the identical weight and Beta (0.5, 0.5) weight, respectively (Table 3). No significant variant segments are identified under Beta (1,25) weight at these thresholds. All of these significant segments belong to the genes that we identified via the genome-wide analysis. However, the *p*-values obtained from these small segments tend to be smaller than the corresponding *p*-value from the larger gene piece analysis. Furthermore, the weight that gives the smallest *p*-value for a large genomic segment is not necessarily the best weight choice for a smaller segment. For example, although gene, COLEC10, was not significant under the Beta (0.5, 0.5) weight, some of the small segments in its gene did show significance with this weight. Finally, we note that for most of the significant segments, the MURAT *p*-values are smaller than the minimum of two SKAT *p*-values obtained by testing LS and FN separately, no matter which weights are chosen.

**Table 3:** Results of analysis of 17 candidate genes using windows of 30 variants.

Chr	Gene	Start Position	End Position	*p*-value		Identified by
				adj.SKAT	MURAT	
*Identical Weight*						
1	WLS	68657154	68661149	2.88 × 10^−7^	2.62 × 10^−6^	SKAT
8	TNFRSF11B	119946373	119949029	1.64 × 10^−7^	8.19 × 10^−8^	Both
8	TNFRSF11B	119955510	119958424	3.31 × 10^−7^	1.66 × 10^−8^	Both
8	TNFRSF11B	119959204	119961438	1.75 × 10^−7^	1.07 × 10^−7^	Both
8	TNFRSF11B	119967247	119969154	1.94 × 10^−7^	2.79 × 10^−8^	Both
8	COLEC10	120002915	120006286	4.55 × 10^−7^	1.66 × 10^−7^	MURAT
8	COLEC10	120006386	120010261	4.22 × 10^−8^	1.11 × 10^−8^	MURAT
8	COLEC10	120010329	120015307	4.42 × 10^−8^	1.92 × 10^−8^	Both
8	COLEC10	120015537	120018127	3.01 × 10^−8^	1.13 × 10^−8^	Both
8	COLEC10	120018159	120021133	3.28 × 10^−8^	1.39 × 10^−8^	Both
8	COLEC10	120021237	120024383	1.97 × 10^−8^	8.22 × 10^−9^	Both
8	COLEC10	120024466	120028106	3.77 × 10^−8^	2.36 × 10^−8^	Both
8	COLEC10	120028696	120031077	2.45 × 10^−8^	8.97 × 10^−9^	Both
8	COLEC10	120031162	120034048	1.84 × 10^−8^	1.16 × 10^−8^	Both
8	COLEC10	120034105	120038678	1.06 × 10^−8^	6.31 × 10^−9^	Both
8	COLEC10	120038959	120040988	1.70 × 10^−8^	8.38 × 10^−9^	Both
8	COLEC10	120041061	120043493	1.89 × 10^−8^	7.56 × 10^−9^	Both
8	COLEC10	120043632	120045339	1.66 × 10^−8^	7.47 × 10^−9^	Both
8	COLEC10	120045484	120048305	3.19 × 10^−8^	1.02 × 10^−8^	Both
8	COLEC10	120048320	120053420	2.26 × 10^−8^	1.29 × 10^−8^	Both
8	COLEC10	120053421	120060026	1.29 × 10^−8^	9.58 × 10^−9^	Both
*Beta(05,05) Weight*						
8	TNFRSF11B	119946373	119949029	5.13 × 10^−8^	5.50 × 10^−8^	Both
8	TNFRSF11B	119959204	119961438	2.52 × 10^−6^	4.74 × 10^−7^	MURAT
8	COLEC10	120006386	120010261	3.22 × 10^−8^	1.18 × 10^−8^	Both
8	COLEC10	120010329	120015307	2.83 × 10^−8^	1.68 × 10^−8^	Both
8	COLEC10	120015537	120018127	1.34 × 10^−8^	1.08 × 10^−8^	Both
8	COLEC10	120028696	120031077	1.09 × 10^−6^	7.32 × 10^−7^	MURAT
8	COLEC10	120045484	120048305	9.63 × 10^−8^	3.22 × 10^−8^	Both

Significant gene segments identified by MURAT or SKAT are shown under different weighting schemes. The significance thresholds for SKAT is 3.42 × 10^−7^, and is 7.51 × 10^−7^ for MURAT. In the table, the adjusted *p*-values for SKAT are defined as the minimum of two *p*-values obtained by testing LS and FN individually.

### Single variant analysis

We also ran a series of single variant analyses for these 17 selected genes (the 3 candidate genes and the additional 14 known BMD-associated genes). In total, 19,325 SNPs are contained in these 17 genes, but at some SNPs, only a few individuals carry the minor allele among the 8810 subjects. Considering the robustness of single variant analysis, we removed SNPs where the minor allele was carried by less than 4 individuals, leaving 19,071 variants for analysis (Supplemental Table 3). Since each test only involves one SNP, no weight choice is required.

There are no significant SNPs if we use 1.2 × 10^−8^, the genome-wide significant threshold used in Zheng *et al*.^16^, as the thresholds for both SKAT and MURAT. However, if we relax the threshold a little to 2 × 10^−8^, then 6 variants in gene COLEC10 (Table 4) are significant and detected by MURAT or SKAT. Analysis either with linear regression or with SKAT gives almost identical results (Supplemental Figure 3) as expected since the score test and the Wald test are asymptotically equivalent.

**Table 4:** Variants identified via single variant analysis with *p*-values less than 2 × 10^−8^.

Chr	Gene	rsID	Position	*p*-values	
				adj.SKAT	MURAT
8	COLEC10	rs7842942	120008587	3.14 × 10^−8^	1.89 × 10^−8^
8	COLEC10	rs1485285	120036138	1.79 × 10^−8^	1.53 × 10^−8^
8	COLEC10	rs7004539	120040499	2.00 × 10^−8^	1.54 × 10^−8^
8	COLEC10	rs6469803	120040538	1.68 × 10^−8^	1.41 × 10^−8^
8	COLEC10	rs12056346	120042191	2.34 × 10^−8^	1.65 × 10^−8^
8	COLEC10	rs4495458	120042780	2.46 × 10^−8^	1.93 × 10^−8^

The adjusted *p*-values for SKAT are defined as the minimum of two *p*-values obtained by testing LS and FN individually.

Similar to results from the small window analysis, in our single variant analyses, the significant SNPs usually show smaller *p*-values than the corresponding ones obtained via set-based analysis. Furthermore, the MURAT *p*-values are much smaller than the adjusted SKAT *p*-values. Hence, the correlation between the phenotypes can be successfully leveraged to gain power through MURAT.

## Discussion

In this paper, by combining individual level phenotype and genotype data from three cohort studies, the Study of Osteoporotic Fractures (SOF), Osteoporotic Fractures in Men (MrOS) Study, and the UK10K project, we investigated the performance of a newly proposed multivariate rare-variant association test (MURAT) through analyses of bone mineral density (BMD). In our investigations, we compared the multivariate test, MURAT, with single-phenotype test, SKAT, by testing BMD at femoral neck (FN) and lumbar spine (LS) jointly and separately for each gene-based variant set. In addition, genes identified via our gene-based analysis and reported from previous literature were further examined by small window and single variant analyses. We also compared performance of three different MAF-based weighting schemes in both MURAT and SKAT with sets of SNPs. Although there is an increasingly large literature on how to best prioritize genetic variants for analysis using external annotation information, particularly in the context of region based tests^8,9^, since our motivation here was to compare single-phenotype approaches with multiple-phenotype approaches, we have not incorporated such external annotations in any of these results.

Despite the potential for improved power associated with multivariate phenotype analysis, all of the genes that we identified as associated with BMD had been previously reported. Indeed, the recent meta-analyses including over 50,000 individuals has identified genetic variants in more than 20 genes as associated with BMD^16,17^. MURAT provides a modest increase in power which cannot compete with the power gain associated with an over 6-fold increase in sample size. To identify new genes where rare genetic variation is associated with BMD, including subjects with more extreme bone phenotypes could be useful. However, MURAT may be of assistance for increasing power for studies involving other phenotypes where it is more challenging to increase the sample size.

Currently, individual level data are required for the calculations in MURAT. Assembling extremely large sample sizes for single variant analysis is sometimes feasible through meta-analysis, where only summary level statistics need to be shared; an extension of SKAT has also been developed that requires only sharing of a set of summary statistics^27^. However, not all analyses can be achieved by sharing only summary statistics; in this collaboration, we were able to create a data set containing individual phenotypes and genotypes for 8810 people, thereby increasing our power as much as is often reasonably possible simply through increased sample sizes for individual-level data. However, since the genetic signals can be very subtle, potential additional power improvements through multivariate analysis are of great interest.

However, it must be acknowledged that a single analysis of all the individual-level data from three studies may be susceptible to bias from confounding arising from different BMD distributions across the cohorts, in conjunction with subtle genetic variation. For example, Table 5 shows that LS and FN were both slightly lower in the SOF study, as would be expected given their female sex and older age. To protect as much as possible against potential confounding, all our analyses were adjusted for sex as well as the top principal components of ancestry, calculated from a subset of analyzed variants with large MAF in the combined dataset. Although, it would probably be better to calculate the PCs from variants not involved in the MURAT tests, i.e. to use variants from non-exonic regions when calculating our PCs, we doubt that this alteration would influence our conclusions comparing test statistics. Furthermore, it could be a worthwhile future endeavor to adapt the MURAT calculations so that they could be performed in a meta-analytic fashion.

**Table 5:** Descriptive statistics, mean (standard deviation), range, skewness, and correlations for BMD phenotypes (before and after log transformation) and covariates in each of the three studies that were combined.

	Mean (standard deviation)			Range		
	MrOS	SOF	UK10K	MrOS	SOF	UK10K
FN	0.78 (0.13)	0.65 (0.11)	0.79 (0.13)	0.27–1.60	0.28 –1.21	0.47–1.18
LS	1.17 (0.25)	0.86 (0.17)	0.99 (0.15)	0.57–2.72	0.44–1.84	0.57–1.84
log FN	−0.11(0.07)	−0.19(0.07)	−0.11(0.07)	−0.56–0.20	−0.56– −0.35	−0.33–0.07
log LS	0.06 (0.09)	−0.07(0.08)	−0.01(0.07)	−0.24–0.43	0.08–0.26	−0.24–0.27
Age	73.9 (5.95)	73.5 (5.26)	52.4 (11.2)	65–100	67–98	17–82
Weight	83.5 (13.0)	67.2 (12.4)	68.1 (12.5)	50.8–136.4	36–132.2	37.9–128.3
	**Skewness**			**Correlation**		
	**MrOS**	**SOF**	**UK10K**	**MrOS**	**SOF**	**UK10K**
FN	0.60	0.61	0.21	0.52	0.60	0.69
LS	1.12	0.66	0.24			
log FN	−0.04	0.01	−0.25	0.53	0.60	0.70
log LS	0.38	0.04	−0.31			

The statistics are calculated based on 1004 women in UK10K project, 3256 women in SOF study, and 4550 men in MrOS study.

Our analysis results clearly show that *p*-values obtained via MURAT are generally smaller than the ones calculated from adjusted SKAT (Supplemental Figures 4–6) no matter which weight matrix is applied, which implies that this multivariate test has the ability to improve power compared with a univariate test in rare-variant association studies. This trend is more clear when we focus on the significant results identified with different analyses. For example, in gene-based analysis, 3 out of 4 significant gene pieces demonstrate smaller MURAT *p*-values (Table 1). Similarly, in small window and single variant analyses, there are 26 out of 28 identified gene segments, and all 6 detected single SNPs where MURAT *p*-values are smaller than the ones obtained via adjusted SKAT, respectively. For gene and small window analyses (Tables 1 and 3), the MURAT *p*-values are generally 30% to 70% less than adjusted SKAT *p*-values, and for single variant analysis (Table 4), the MURAT *p*-values can be 15% to 40% less than adjusted SKAT *p*-values.

To further investigate the relationship between *p*-values and number of variants contained in the regions, we categorized all 24,333 gene pieces into 4 groups based on the number of variants in the gene piece. Specifically, group 1 includes 1360 pieces that contain less than 100 variants; group 2 contains 11720 pieces that have 100 to 500 variants; group 3 has 8670 pieces which contain 500 to 1000 variants; and the remaining 2583 pieces belong to group 4, since they all have more than 1000 SNPs. By applying the F-test from an analysis of variance, we then compared the *p*-values obtained via MURAT with identical weight between these groups, and obtained evidence of strongly-different *p*-value distributions (*p*-value = 2 × 10^−16^). Tukey’s test for multiple comparisons showed that groups having more variants generally had larger *p*-values. The box plots for *p*-values within each group, and plots of 95% family-wise confidence levels for Tukey’s multiple comparison procedure are shown in Supplemental Figures 7 and 8. This result demonstrates that a properly defined set plays an important role in set-based testing methods. Therefore, in addition to genome-wide gene-based analysis of the entire genic regions, we also performed small window and single variant analyses for some well-replicated BMD-associated genes to avoid the situation when limited number of associated variants exist in large region the set-based methods cannot distinguish signal from noise.

In all of our analyses, we directly combined individual level data from the three studies. Since there are some differences across studies in ascertainment strategies, covariate measurements and platforms for sequencing/genotyping, it could be argued that “study” itself may play as an important covariate and needs to be adjusted for in the association tests. Hence, for the 17 selected genes listed in Supplemental Table 3, we repeated our analyses with MURAT and SKAT including study indicator variables, with the three different weights. As can be seen in Supplemental Table 4, inclusion of study indicators results in very little change in results, and the same genes would be called significant. Moreover, the *p*-values obtained via two models, with or without study covariates, are almost the same (Supplemental Figure 9). Hence, we infer that if we had added a study indicator to all analyses, our final results would have been almost identical.

Additional information, such SNPs annotation, can be used for refining subsets/windows^28^. However, the choice of an optimal size of the window to be analyzed remains a considerable issue. For instance, loss of power can be significantly affected by noise-to-signal ratio of a SNPs set. Thus, some studies have shown that the optimal size depends on the test statistic chosen and the proportion of causal variants^8^, and others have proposed to optimize the window size^29^. Alternatively, one can attribute weights for different class of variants by up-weighting functionally damaging variants. The bioinformatics algorithms^30–32^ can produce inaccurate predictions, however, they should be considered as just one possible choice for refining windows; there is a need to develop high-throughput functional characterization methods.

Our analyses clearly show that the multivariate method has significant potential to improve power in rare variant association studies for both set-based and single variant analyses. Since in many large-scale sequencing studies, multiple measurements of related traits are available for investigation, a multivariate method is well-worth considering to boost power, especially when there are pleiotropic effects or strongly-correlated phenotypes (for example ρ >0.6).

In our analyses, we used three different weights, and the identical weight showed better results. The choice of weighting scheme is critical to improve test power. Although there are a few publications that have evaluated multiple weights in different study designs through simulation^33,34^, there is no strong consensus on ideal weight choice, and of course it will depend on the true genetic architecture at the locus. It may be worth additional investigation to understand which weights retain the most power even when the true genetic model is incorrectly modelled. In addition, similar to other set-based association studies, here we have only provided an overall *p*-value for the association between a group of variants and the phenotypes. We note also that a nonparametric multiple-trait set-based test has recently been developed based on generalized similarity U-statistics^35^, and it would be worth comparing its performance with MURAT.

Once a new association is found, it becomes interesting to ask which individual variants drive the association and to estimate corresponding genetic parameters. Careful fine-mapping^36^ and follow-up replication studies^8^ are usually needed to achieve these goals. Nevertheless, a few methods for variable selection or adaptive combinations of *p*-values have been proposed to fill the gap between SNP set analysis and fine mapping of individual variants^37–39^. Investigations of how such techniques perform for a multivariate test such as MURAT would be worthwhile.

## Methods

### Data and quality control methods

In the analyses, we combined individual level autosomal data from three studies.

The Study of Osteoporotic Fractures (SOF)^19^ (http://sof.ucsf.edu/interface/) is a prospective multicentre study that enrolled 9704 women over age 65^40–42^, and the Osteoporotic Fractures in Men study (MrOS) is a multicentre study of musculoskeletal health in men 65 and older^20,21^ that enrolled 5,994 participants. In both studies, participants were required to be able to walk without assistance at study entry. Written informed consents were obtained for both studies, and Institutional Review Boards at each enrollment site approved these studies. Genotyping for both SOF and MrOS samples was performed with the Illumina HumanOmni1_Quad_v1-0 H array. Samples with call rate under 97% were excluded, as were samples with evidence for non-European global ancestry or showing unusual heterozygosity or relatedness^16^. Genotypes with MAF < 0.01 or Hardy-Weinberg disequilibrium (at *p* < 1e-4) were excluded, and the remaining genotypes were subsequently imputed against the combined UK10K/1000Genomes reference panel. Singletons were excluded from the reference panels prior to merging, and variants missing from one reference were imputed into the other before creating the combined reference. Genotypes were pre-phased without using the reference panel, and then variants from the reference were imputed into the best-guess haplotypes. Variants with INFO score less than 0.4 were excluded. Full details of the imputation pipeline can be found in^16^.

There were 3,781 individuals who underwent whole genome sequencing in the UK10K study^18^ (http://www.uk10k.org/ and www.gefos.org). These individuals were selected from participants in the TwinsUK study (http://www.twinsuk.ac.uk/) or the ALSPAC study (http://www.bristol.ac.uk/alspac/) and had previously been assessed for BMD^43–45^. Whole genome sequencing was performed with the Illumina HiSeq platform with an average read depth of 6.7. Identified variants were quality filtered with a VQSLOD score of −0.6804, Hardy-Weinberg equilibrium p-value < 1e-6, or evidence of batch effects^16^. Imputation was performed against a combined reference panel formed by merging the 1000 Genome haplotypes with UK10K haplotypes, removing singletons, and imputing missing variants from each of the two studies into the other^16^. Data were pre-phased without a reference panel, and then imputed with IMPUTE2 using an info score of 0.4 to remove poorly imputed variants^16,18^.

### BMD and covariate measurements

In the MrOS study, BMD at the femoral neck and lumbar spine was measured using dual-energy X-ray absorptiometry (DXA) with Hologic QDR‐4500 W scanners (Hologic, Inc., Bedford, MA, USA) at the baseline clinic visit. A central quality‐control laboratory, certification of DXA technicians, and standardized procedures for scanning were implemented to ensure reproducibility of DXA measurements. At baseline, a hip phantom was circulated and scanned at the six clinical centers. Cross‐calibration studies indicated no linear differences across scanners, and the interscanner coefficient of variation (CV) was 0.9%. Height was measured using a Harpenden stadiometer, and weight was measured by a standard balance beam or an electric scale. Two consecutive height measurements were taken, and the average of these measurements was used in the analysis. If the two height measurements differed by >5 mm, a third measurement was performed, and the average of the two values with the smallest difference was used in the analysis.

In the SOF, BMD of the proximal femur and spine was measured using DXA (QDR‐1000, Hologic) during a follow up visit between 1989 and 1990. Densitometry quality control methods have been previously published^41,42^. Briefly, paired initial and follow up hip scans were analyzed using the automated “compare” feature of the Hologic software. Quality control center technicians reviewed a random sample. In addition, all scans identified by the technicians for certain problems such as changes in positioning between the initial and follow up scans or difficulty defining bone edges were reviewed at the quality control center. An anthropometric spine phantom was scanned daily, and a hip phantom was scanned once per week at each clinic to assess longitudinal performance of the scanners. Weight was measured using a standard balance beam scale, and height was measured using a Harpenden stadiometer.

In the UK10K study, lumbar spine and femoral neck BMD measurements were obtained from the available measurements taken in the ALSPAC and Twins UK studies. Specifically, BMD was measured using DXA with Hologic QDR 4500 W for TwinsUK participants, and in ALSPAC, TB-DXA scans were performed on all participants, using a Lunar Prodigy scanner (Lunar Radiation Corp, Madison, WI) with paediatric scanning software (GE Healthcare Bio-Sciences Corp., Piscataway, NJ). In ALSPAC, all DXA scans were subsequently reviewed by a trained researcher, and re-analysed as necessary, to ensure that borders between adjacent ROI’s were placed correctly by the automated software. Data were standardized within the two source studies prior to combining. Both TwinsUK and ALSPAC use Harpenden stadiometer to measure height.

For 3256 women in SOF and 4550 men from MrOS with imputed genotype data, and 1004 sequenced women from the UK10K study, BMD measures were also available. With these 3 cohorts together, there are 8810 subjects included in our analyses.

We considered two correlated bone density phenotypes, BMD at femoral neck (FN) and lumbar spine (LS). A log transformation was applied to these two phenotypes before the analyses so that they have bell-shaped distributions, and after the log transformation the correlation between the phenotypes in combined data set is 0.6775. In all analyses, variables age, age^2^, gender, and weight were used as covariates. In addition, the first five principal components (PCs), obtained using a subset of genotype data that were randomly selected from our analyzed variants with large MAF (>0.48) and low LD (r^2^ < 0.1) based on all 8810 subjects, were also included as covariates to adjust for population structure.

Descriptive statistics for BMD and covariate measurements are listed in Table 5, separately for the three studies. The expected lower BMD in females can be seen for the SOF study relative to MrOS; this is not only due to sex, but also because they are older than the men in MrOs. Mean values of BMD from the UK10K study are similar to the MrOS study despite the fact that we used only sequencing data from women; however, the average participant age was younger in UK10K.

### Definition of gene pieces

Many previous studies related with BMD association have focused on common variants (MAF >0.05), and have insufficiently assessed the role of low frequency (MAF 0.01–0.05) and rare (MAF <0.01) genetic variation (Zheng *et al*.^16^). Hence, we used only the rare variants in or near genes on the autosomes (excluding chromosomes X and Y) analyzed in Zheng *et al*.^16^, where rare variants were defined as SNPs with MAF <0.05, and the MAFs were estimated based on the 21 cohorts used in their paper. However, since only three out of the 21 cohorts are being analyzed here, some of the variants we used may have larger MAF than 0.05. The majority of analyzed variants are still rare: 76.67% have MAF <0.05 and 81.58% have estimated MAF <0.10.

Variants were included in a gene region if they fell within a window defined by the maximal extent of all isoforms of a gene, with an additional 5 kb on either end of the maximal extent of all isoforms. In addition, for genes that span a distance greater than 150 kb, the segments were subdivided into multiple non-overlapping smaller pieces of at most 100 kb and at least 50 kb. We refer to these genomic segments that were used in the region-based analyses as *gene pieces*; Supplemental Table 1 shows the number of gene pieces on each chromosome. The number of variants in the gene pieces is extremely variable and ranges from just one variant to a maximum of 2898 SNPs (Supplemental Figure 1).

### Regions for analysis of candidate genes

For more in depth investigations of the performance of multivariate analysis methods, we selected several candidate genes and divided their genetic data into smaller regions. Candidate genes were selected in two ways, firstly we selected the genes with the most significant results of the “gene-piece” analyses, and secondly, we analyzed genes that have well-replicated SNP associations with bone mineral density^17^. For both sets of genes, we divided the gene pieces into smaller, non-overlapping segments containing at most 30 variants. We also performed single variant analyses of these two sets of candidate genes. The number of regions analyzed for each of the two sets of candidate genes is shown in Supplemental Table 3.

### Test statistics

For a univariate method that tests the two BMD phenotypes separately, we used SKAT to identify association. For a joint multivariate analysis of the two BMD phenotypes simultaneously, we used MURAT^12^. For the region-based tests, we compared three contrasting different strategies for weighting the variants within a region. We first assigned identical weights to all variants. Secondly, we used a beta distribution, $\sqrt{{w}_{j}=Beta(MA{F}_{j};{a}_{1},{a}_{2})}$ to specify weights due to its flexibility of accommodating a wide range of scenarios^3^. For the two parameters of the beta distribution, we considered two situations: (i) a_1_ = 1 and a_2_ = 25, (the parameters suggested by SKAT^4^). These choices allow higher weights for the rare variants (with MAF <1%) and give modest weights for low frequency variants with MAF 1–5%. (ii) a_1_ = a_2_ = 0.5, which is equivalent to $\sqrt{{w}_{j}}=1/\sqrt{MA{F}_{j}(1-MA{F}_{j})}$ for the *j*th variant. This choice puts more weight onto the very rare variants and almost zero weights to variants with MAF >1%^4,46^.

### Brief description of MURAT

A description of this method is found in more detail in Sun *et al*.^12^. The method performs a joint analysis of more than one phenotype, and tests for association simultaneously for all phenotypes and all variants in a region.

Suppose for the *i* th subject, *i* = 1, …, *N*, we observe *K* correlated continuous phenotypes, **Y**_i_ = (*y*_*i*1_, *y*_*i*2_, …, *y*_*iK*_)^*T*^; *m* covariates, **X**_i_ = (*x*_*i*1_, *x*_*i*2_, …, *x*_*im*_)^*T*^; and genotype values for a group of *v* variants, **G**_i_ = (*g*_*i*1_, *g*_*i*2_, …, *g*_*iv*_)^*T*^, where *g*_*ij*_ for *j* = 1*, …, v* are coded as 0, 1, or 2, representing the number of minor alleles that individual *i* carries for the *j* th variant. Similar to SKAT^4^, a single phenotype, *Y*_*ik*_, is linked with *G*_*i*_ and *X*_*i*_ via a linear mixed model

1 $${Y}_{ik}={\alpha }_{0k}+{{\boldsymbol{\alpha }}}_{k}^{T}{{\boldsymbol{X}}}_{i}+{{\boldsymbol{\beta }}}_{k}^{T}{{\boldsymbol{G}}}_{i}+{\varepsilon }_{ik}\,{\rm{for}}\,\,k=1,\ldots ,K$$

where *α*_0*k*_ is a scalar for intercept; ***α***_*k*_ and ***β***_*k*_ are corresponding coefficient vectors, and ***β***_*k*_ ~ *N*(**0**, *τ* ^2^ *W*) is a vector of random effects, such that *τ* ^2^ is an unknown variance component, *W* = *diag*{*w*_1_, …, *w*_*v*_} is a weight matrix with *w*_*j*_ representing the weight for the *j* th variant; and *ε*_*ik*_ is random error following a standard normal distribution.

However, since *K* phenotypes are correlated, in MURAT, a multivariate linear mixed model is used to jointly model these phenotypes,

2 $${{\boldsymbol{Y}}}_{i}={{\boldsymbol{\alpha }}}_{0}+({{\bf{1}}}_{K}\otimes {{\boldsymbol{X}}}_{i}^{T}){\boldsymbol{\alpha }}+({{\bf{1}}}_{K}\otimes {{\boldsymbol{G}}}_{i}^{T}){\boldsymbol{\beta }}+{{\boldsymbol{\varepsilon }}}_{i},$$

where ***α***_0_ = (*α*_01_, …, *α*_0*K*_)^*T*^, ${\boldsymbol{\alpha }}={({{\boldsymbol{\alpha }}}_{1}^{T},\ldots ,{{\boldsymbol{\alpha }}}_{K}^{T})}^{T}$, ${\boldsymbol{\beta }}={({{\boldsymbol{\beta }}}_{1}^{T},\ldots ,{{\boldsymbol{\beta }}}_{K}^{T})}^{T}$, ***ε***_*i*_ = (*ε*_*i*1_, *…*, *ε*_*iK*_)^*T*^, **1**_*K*_ is a K-dimensional vector of ones, and ⊗ represents Kronecker product. In this multivariate model, the variant effect, ***β***, is assumed to follow a multivariate normal distribution, *N*(**0**, Σ_*β*_). In addition, to account for trait pleiotropy effects, ***β***_*k*_ and ${{\boldsymbol{\beta }}}_{k^{\prime} }$, for *k* ≠ *k*′, are assumed to be correlated, such that $Corr({\beta }_{kj},{\beta }_{k^{\prime} j})=\rho$ for variant j with *ρ* unknown, and $Corr({\beta }_{kj},{\beta }_{k^{\prime} j^{\prime} })=0$ for variant j and variant j’. That is, MURAT assumes a common correlation for the effects of the same variant on different phenotypes, but the effects of different variants are uncorrelated. Consequently, the covariance matrix *Σ*_*β*_ = *τ* ^2^ *R*⊗*W*, where $R\mathrm{=(1}-\rho ){I}_{K}+\rho {{\bf{1}}}_{K}{{\bf{1}}}_{K}^{T}$. Moreover, to account for the correlation beween phenotypes, the model error term, ***ε***_*I*_ is assumed to independently follow a multivariate normal distribution *N*(**0**,Σ), where Σ is an arbitrary symmetric and positive definite matrix.

In order to test the association between genotype and multiple phenotypes, a score type statistic was derived to test whether ***β*** equals zero in MURAT. By using a data-adaptive procedure to manage unknown correlation, *ρ*, among variant effects, ***β***_*k*_’s, MURAT can obtain *p*-values analytically via a one dimensional numerical integration. It has been shown^12^ that by incorporating arbitrary correlations among multiple phenotypes and considering the existence of trait pleiotropy effects, the proposed MURAT has the potential to increase test power compared with other univariate tests. In addition, since MURAT calculated *p*-values analytically without permutation or resampling, MURAT can be used for a genome-wide analysis.

## Electronic supplementary material

Supplemental Material
