# Supplementary material for: Exome-wide rare variant analyses of two bone mineral density phenotypes: the challenges of analyzing rare genetic variation
Source: Sci Rep. 2018 Jan 9;8:220. doi: 10.1038/s41598-017-18385-9 (PMC5760616; doi:10.1038/s41598-017-18385-9)

**Supplemental Material for**

Exome-wide rare variant analyses of two bone mineral density phenotypes: the challenges of analyzing rare genetic variation

Jianping Sun^1,2^, Karim Oualkacha^3^, Vincenzo Forgetta^2^, Hou-Feng Zheng^4,5^, J. Brent Richards^1,2,6^, Daniel S. Evans^7^, Eric Orwoll^8^, and Celia M.T. Greenwood^1,2,6,9,*^.

1. Department of Epidemiology, Biostatistics and Occupational Health, McGill University,

Montreal, QC, Canada.

2. Lady Davis Institute for Medical Research, Jewish General Hospital, Montreal, QC, Canada.

3. Département de mathématiques, Université du Québec à Montréal, Montreal, QC, Canada.

4. Institute of Basic Medical Sciences, Westlake Institute for Advanced Study, Westlake

University, Hangzhou, Zhejiang, China

5. Institute of Aging Research, School of Medicine, Hangzhou Normal University, Hangzhou,

Zhejiang, China

6. Department of Human Genetics, McGill University, Montreal, QC, Canada.

7. California Pacific Medical Center Research Institute, San Francisco, CA, US

8. Department of Medicine, Bone and Mineral Unit, Oregon Health and Science University,

Portland, OR, USA

9. Department of Oncology, McGill University, Montreal, QC, Canada.

**Table of Contents**

**Supplemental Text**  **Pages 3-4**

Table S0: Correlations among permutated *p*-values under three weights.

**Supplemental Tables**  **Pages 5-8**

Supplemental Table 1: The number of gene pieces and whole genes analyzed on each chromosome.

Supplemental Table 2: The *p*-values obtained from gene-based analysis for 14 well-replicated bone genes.

Supplemental Table 3: Number of regions and number of SNPs used in analysis of each candidate gene, in the small window analysis and in single variant analysis.

Supplemental Table 4: Significant genes identified via models with and without study covariates.

**Supplementary Figures Pages 9-17**

Supplemental Figure 1: Boxplots of number of SNPs in each gene piece, by chromosome.

Supplemental Figure 2: Scree plot displaying the variability explained by principal components calculated using 6289 SNPs.

Supplemental Figure 3: *p*-value comparisons between SKAT and linear regression in single variant analysis.

Supplemental Figure 4: *p*-value comparisons between SKAT and MURAT for gene-based analysis with three different weights.

Supplemental Figure 5: *p*-value comparisons between SKAT and MURAT for small window analysis with three different weights.

Supplemental Figure 6: *p*-value comparisons between SKAT and MURAT in single variant analysis, where the adjusted SKAT *p*-value is defined as the minimum of two *p*-values obtained by testing LS and FN individually.

Supplemental Figure 7: Box plot of *p*-values, obtained via MURAT with identical weight for gene pieces analysis, within different groups.

Supplemental Figure 8: 95% family-wise confidence level for Tukey’s multiple comparison of mean *p*-values from MURAT analyses of gene pieces grouped by the number of SNPs in the gene pieces.

Supplemental Figure 9: For 17 selected genes listed in Supplementary Table 3, *p*-values are compared between models with and without study indicator covariates.

**Supplemental Text**

**Estimate exome-wide significant threshold**

To decide on appropriate significance thresholds in this analysis, we performed permutations of the two phenotypes versus the genotypes and repeated the region-based analyses on 500 contiguous gene pieces on chromosome 9. It would not have been computationally feasible to perform genome-wide permutations for all the different test statistics and weights that were used. For SKAT, 500 permutations were conducted for each gene piece under three different weights. Due to the computational cost, for MURAT, only 200 permutations were performed for each gene piece, using identical weights, and additionally 10 permutations per gene piece were conducted with Beta (1, 25) and Beta (0.5, 0.5) weights, respectively. Using the extrapolation method described in Xu et al. (2014) together with some adaptations as described below, we can estimate genome-wide thresholds based on these sets of permuted *p*-values from a subregion of the genome.

For SKAT, the thresholds for each weight can be calculated by applying Xu’s method on minimum permuted *p*-values obtained across the two phenotypes. Specifically, the thresholds thus obtained are 6.10x10^-7^ for identical weight, 4.53x10^-7^ for Beta (1,25) weight, and 2.44x10^-7^ for Beta (0.5,0.5) weight, respectively. Furthermore, an overall estimated significance threshold for SKAT can be obtained by looking at the minimum *p*-values across all three weights and two phenotypes; the extrapolation method in this case gives a threshold of 1.12x10^-7^. This estimate is, in fact, smaller than a Bonferroni threshold (0.05/(3 weights by 2 phenotypes by 24,333 genes) = 3.42x10^-7^. Since we would expect an estimated threshold that takes correlation into account to be *less* stringent than Bonferroni, we propose to use the Bonferroni threshold to establish significance for SKAT.

Similarly, we estimated the threshold for MURAT with identical weight at 2.89x10^-6^. However, because only 10 MURAT permutations were performed under Beta (1, 25) and Beta (0.5, 0.5) weights, the overall threshold for MURAT, across the 3 weights, cannot be obtained using the extrapolation method as for SKAT. Instead, we compared two approximations. Firstly, we approximated the significance threshold by using methods developed to estimate the number of independent SNP tests based on a matrix of correlations. Firstly, we estimated the number of independent tests based on the correlation matrix between MURAT permutated p-values under three different weights (Greenwood et al. 2015). For example, by using estimation methods proposed by Cheverud (2001) and Li and Ji (2005), we obtained the number of independent tests between the 3 weights as 1.95 and 2.38, respectively. If we compromise between these 2 values, and set the number of independent test as 2.2, then we can adjust the identical weight MURAT threshold by this number, and then back-transform from the fitted regression line to obtain the desired overall MURAT threshold at 1.36x10^-6^. We also took a second approach. Noticing that the correlations between SKAT permutated *p*-values and MURAT permuted *p*-values are similar under three different weights (Table S0), we can calculate an inflation factor from the SKAT estimated thresholds as 1.12x10^-7^ / ( (6.10x10^-7^+4.53x10^-7^+2.44x10^-7^)/3 )= 0.26, and then inflate the overall MURAT genome-wide prediction from the threshold for identical weight by this amount. That is, the estimated overall MURAT threshold becomes 2.89x10^-6^x0.26=7.51x10^-7^. Of these two estimates, we therefore propose to use the more stringent threshold here of 7.51x10^-7^ for significance of MURAT.

**Table S0:** Correlations among permutated *p*-values under three weights. For SKAT, the minimum *p*-values between two phenotypes are used. Correlations are based on 10 permutations for MURAT and the results of SKAT using the same 10 permuted data.

|  | SKAT | MURAT |
| --- | --- | --- |
| Identical and Beta(1,25) | 0.23 | 0.22 |
| Identical and Beta(0.5,0.5) | 0.78 | 0.77 |
| Beta(1,25) and Beta(0.5,0.5) | 0.52 | 0.52 |

**References:**

1. Xu, C., Tachmazidou, I., Walter, K., Ciampi, A., Zeggin, E., Greenwood, C., and the UK10K Consortium. (2014) Estimating Genome-Wide Significance for Whole-Genome Sequencing Studies. *Genetic Epidemiology*;38(4):281-90. doi: 10.1002/gepi.21797. Epub 2014 Feb 14.

2. Greenwood, C.M.T., Xu, C., Ciampi, A. (2015). Significance thresholds for rare variant signals. 169-183. In *Assessing rare variation in complex traits: design and analysis of genetic studies*. Editors: Andrew Morris, Eleftheria Zeggini. Springer, New York. DOI: 10.1007/978-1-4939-2824-8.

3. Cheverud JM (2001) A simple correction for multiple comparisons in interval mapping genome scans. Heredity (Edinb) 87(Pt 1):52–58.

4. Li J, Ji L (2005) Adjusting multiple testing in multilocus analyses using the eigenvalues of a

correlation matrix. Heredity 95(3):221–227

**Supplementary Tables:**

**Supplemental Table 1:** The number of gene pieces and whole genes analyzed on each chromosome.

| Chr | # gene pieces | # genes | Chr | # gene pieces | # genes | Chr | # gene pieces | # genes |
| --- | --- | --- | --- | --- | --- | --- | --- | --- |
| 1 | 2,437 | 2,033 | 9 | 997 | 787 | 17 | 1,298 | 1,180 |
| 2 | 1,720 | 1,259 | 10 | 1,049 | 753 | 18 | 436 | 285 |
| 3 | 1,578 | 1,065 | 11 | 1,560 | 1,300 | 19 | 1,485 | 1,451 |
| 4 | 1,081 | 737 | 12 | 1,312 | 1,053 | 20 | 655 | 556 |
| 5 | 1,223 | 887 | 13 | 492 | 327 | 21 | 299 | 239 |
| 6 | 1,403 | 1,042 | 14 | 807 | 640 | 22 | 505 | 448 |
| 7 | 1,287 | 897 | 15 | 776 | 599 |  |  |  |
| 8 | 977 | 675 | 16 | 956 | 839 |  |  |  |

**Supplemental Table 2:** The *p*-values obtained from gene-based analysis for 14 well-replicated bone genes. The adjusted *p*-value for SKAT is defined as the minimum of two *p*-values obtained by testing LS and FN individually.

|  |  |  | *p*-values | | | | | |
| --- | --- | --- | --- | --- | --- | --- | --- | --- |
|  |  |  | Identical weight | | Beta (1,25) weight | | Beta (0.5,0.5) weight | |
| Chr | Gene | # of SNPs | adj.SKAT | MURAT | adj.SKAT | MURAT | adj.SKAT | MURAT |
| 4 | SPP1 | 163 | 5.24x10^-2^ | 5.79x10^-2^ | 1.42x10^-1^ | 2.99x10^-1^ | 3.51x10^-2^ | 3.87x10^-2^ |
| 6 | RUNX2.1 | 895 | 1.93x10^-1^ | 5.36x10^-3^ | 3.31x10^-1^ | 5.45x10^-1^ | 3.56x10^-1^ | 5.24x10^-2^ |
| 6 | RUNX2.2 | 813 | 2.25x10^-1^ | 7.45x10^-2^ | 5.15 x10^-1^ | 8.49x10^-1^ | 4.42x10^-1^ | 2.64x10^-1^ |
| 6 | RUNX2.3 | 1508 | 1.87x10^-1^ | 4.79x10^-1^ | 5.36x10^-1^ | 9.40x10^-1^ | 1.89x10^-1^ | 5.47x10^-1^ |
| 6 | RSPO3 | 636 | 4.09x10^-2^ | 1.34x10^-1^ | 9.40x10^-2^ | 2.43x10^-1^ | 4.71x10^-2^ | 1.29x10^-1^ |
| 6 | ESR1.1 | 946 | 5.31x10^-6^ | 4.07x10^-5^ | 5.54x10^-1^ | 8.13x10^-1^ | 4.29x10^-6^ | 1.77x10^-5^ |
| 6 | ESR1.2 | 920 | 6.82x10^-3^ | 1.49x10^-2^ | 2.80x10^-1^ | 4.96x10^-1^ | 2.16x10^-2^ | 4.48x10^-2^ |
| 6 | ESR1.3 | 838 | 6.18x10^-2^ | 3.72x10^-1^ | 3.57x10^-1^ | 8.43x10^-1^ | 1.26x10^-1^ | 6.66x10^-1^ |
| 6 | ESR1.4 | 1005 | 5.23x10^-1^ | 9.89x10^-1^ | 4.50x10^-1^ | 8.72x10^-1^ | 5.76x10^-1^ | 8.86x10^-1^ |
| 6 | ESR1.5 | 863 | 1.63x10^-1^ | 5.90x10^-1^ | 1.95x10^-2^ | 1.09x10^-1^ | 9.78x10^-2^ | 3.31x10^-1^ |
| 7 | WNT16 | 221 | 8.36x10^-5^ | 8.37x10^-5^ | 4.69x10^-2^ | 6.01x10^-2^ | 9.47x10^-4^ | 4.07x10^-4^ |
| 10 | DKK1 | 128 | 2.41x10^-1^ | 1.66x10^-1^ | 5.82x10^-2^ | 6.33x10^-3^ | 1.45x10^-1^ | 1.75x10^-2^ |
| 11 | SOX6.1 | 767 | 3.43x10^-1^ | 6.03x10^-1^ | 8.57x10^-1^ | 9.99x10^-1^ | 5.44x10^-1^ | 7.45x10^-1^ |
| 11 | SOX6.2 | 719 | 3.73x10^-1^ | 8.81x10^-1^ | 7.98x10^-1^ | 9.99x10^-1^ | 2.75x10^-1^ | 7.51x10^-1^ |
| 11 | SOX6.3 | 777 | 1.20x10^-3^ | 1.08x10^-2^ | 6.48x10^-1^ | 7.90x10^-1^ | 6.33x10^-4^ | 7.97x10^-3^ |
| 11 | SOX6.4 | 705 | 5.98x10^-3^ | 5.98x10^-3^ | 1.88x10^-1^ | 7.21x10^-1^ | 5.39x10^-3^ | 8.62x10^-3^ |
| 11 | SOX6.5 | 812 | 1.41x10^-1^ | 2.89x10^-1^ | 2.18x10^-1^ | 6.10x10^-1^ | 1.54x10^-1^ | 2.22x10^-1^ |
| 11 | SOX6.6 | 803 | 7.00x10^-2^ | 2.26x10^-1^ | 4.98x10^-1^ | 7.38x10^-1^ | 2.02x10^-1^ | 3.69x10^-1^ |
| 11 | SOX6.7 | 726 | 6.34x10^-2^ | 2.30x10^-1^ | 5.05x10^-1^ | 7.93x10^-1^ | 1.95x10^-1^ | 4.48x10^-1^ |
| 11 | SOX6.8 | 531 | 2.11x10^-1^ | 3.46x10^-1^ | 3.82x10^-1^ | 7.65x10^-1^ | 2.73x10^-1^ | 5.06x10^-1^ |
| 11 | LRP4 | 482 | 8.54x10^-2^ | 1.21x10^-1^ | 9.23x10^-2^ | 3.61x10^-1^ | 2.20x10^-1^ | 1.92x10^-1^ |
| 11 | LRP5 | 720 | 3.92x10^-3^ | 3.64x10^-5^ | 2.77x10^-1^ | 5.03x10^-1^ | 4.38x10^-3^ | 4.08x10^-5^ |
| 12 | PTHLH | 194 | 4.19x10^-2^ | 1.89x10^-1^ | 2.95x10^-1^ | 5.77x10^-1^ | 1.53x10^-1^ | 3.78x10^-1^ |
| 12 | SP7 | 188 | 3.16x10^-4^ | 1.36x10^-3^ | 3.17x10^-1^ | 5.44x10^-1^ | 1.53x10^-4^ | 4.65x10^-4^ |
| 13 | TNFSF11 | 540 | 3.43x10^-2^ | 8.52x10^-2^ | 3.28x10^-2^ | 1.18x10^-1^ | 9.93x10^-3^ | 3.78x10^-2^ |
| 17 | SOST | 135 | 1.05x10^-4^ | 1.42x10^-4^ | 7.16x10^-2^ | 2.32x10^-1^ | 4.15x10^-5^ | 1.30x10^-4^ |
| 17 | SOX9 | 124 | 7.62 x10^-1^ | 8.76x10^-1^ | 7.44x10^-1^ | 8.35x10^-1^ | 4.94x10^-1^ | 6.91x10^-1^ |

**Supplemental Table 3**: Number of regions and number of SNPs used in analysis of each candidate gene, in the small window analysis and in single variant analysis.

| Chr | Gene | # of regions in small window analysis | # of SNPs in single variant analysis |
| --- | --- | --- | --- |
| 1 | WLS | 32 | 930 |
| 4 | SPP1 | 6 | 162 |
| 6 | RSPO3 | 22 | 635 |
| 6 | ESR1 | 154 | 4528 |
| 6 | RUNX2 | 109 | 3180 |
| 7 | WNT16 | 8 | 219 |
| 8 | TNFRSF11B | 13 | 373 |
| 8 | COLEC10 | 29 | 848 |
| 10 | DKK1 | 5 | 128 |
| 11 | LRP5 | 24 | 719 |
| 11 | LRP4 | 17 | 471 |
| 11 | SOX6 | 198 | 5711 |
| 12 | PTHLH | 7 | 192 |
| 12 | SP7 | 7 | 187 |
| 13 | TNFSF11 | 18 | 531 |
| 17 | SOST | 5 | 135 |
| 17 | SOX9 | 5 | 122 |

**Supplemental Table 4**: Significant genes identified via models with and without study covariates. The significance levels are 7.51x10^-7^ for MURAT and 3.42x10^-7^ for SKAT. Since SOF only included women, MrOS only included men, and only data from sequenced women in UK10K were used, we included 3 indicator variables capturing study and sex, and we excluded the overall intercept to ensure identifiability. Inclusion of study indicators results in very little change in the results, and the same genes would be called significant.

|  |  | Without study covariate | | | With study covariates | | | |
| --- | --- | --- | --- | --- | --- | --- | --- | --- |
| Chr | Gene | adj.SKAT | | MURAT | adj.SKAT | | | MURAT |
|  |  |  | Identical weight | | |  | |  |
| 1 | WLS | 8.91x10^-8^ | | 4.74x10^-7^ | 8.08x10^-8^ | | | 6.87x10^-7^ |
| 8 | TNFRSF11B | 5.72x10^-7^ | | 1.36x10^-7^ | 6.05x10^-7^ | | | 1.49x10^-7^ |
| 8 | COLEC10 | 1.92x10^-8^ | | 1.51x10^-8^ | 2.74x10^-8^ | | | 2.06x10^-8^ |
|  |  |  | | Beta(0.5,0.5) | | |  |  |
| 8 | TNFRSF11B | 2.66x10^-6^ | | 2.07x10^-7^ | 3.31x10^-6^ | | | 2.46x10^-7^ |

**Supplementary Figures:**

**Supplemental Figure 1:** Boxplots of number of SNPs in each gene piece, by chromosome.


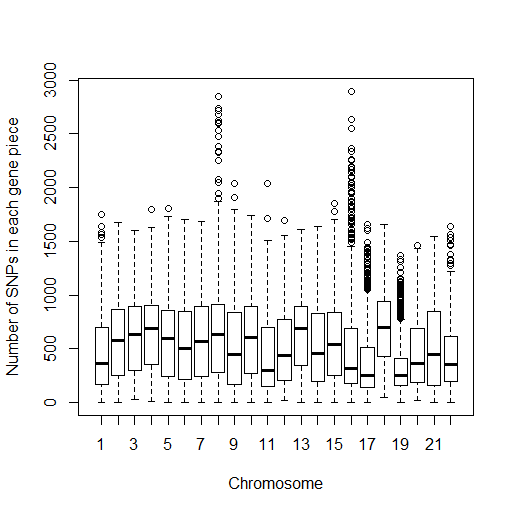


**Supplemental Figure 2:** Scree plot displaying the variability explained by principal components calculated using 6289 SNPs


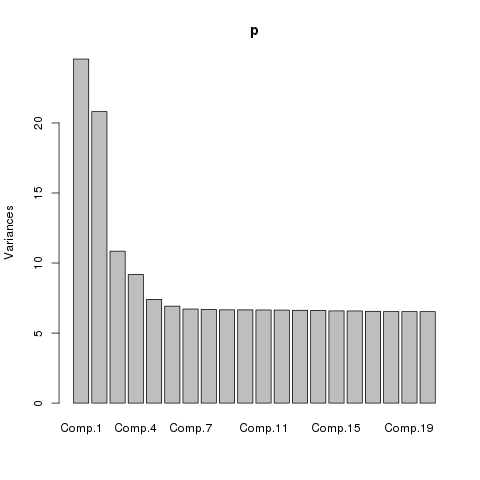


**Supplemental Figure 3:** *p*-value comparisons between SKAT and linear regression in single variant analysis.


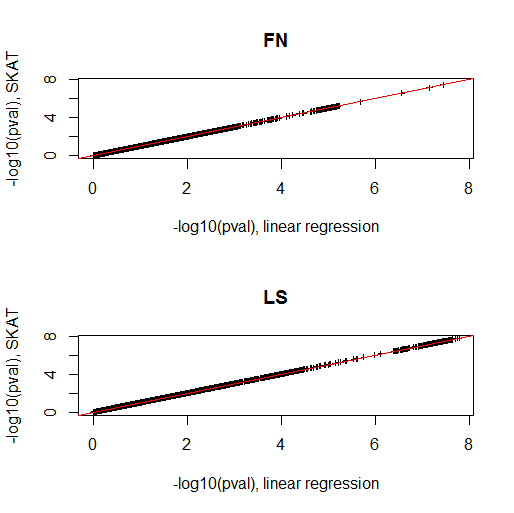


**Supplemental Figure 4:** *p*-value comparisons between SKAT and MURAT for gene-based analysis with three different weights. The adjusted SKAT p-value is defined as the minimum of the two *p*-values obtained by testing LS and FN individually. **A:** identical weights. **B:** Weights based on the Beta (1,25) distribution. **C.** Weights based on Beta (0.5, 0.5).


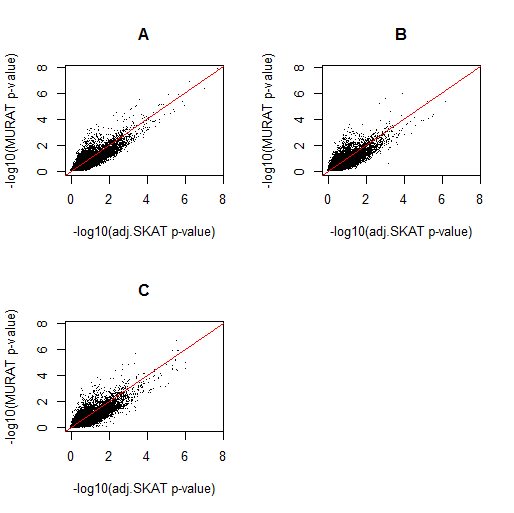


**Supplemental Figure 5:** *p*-value comparisons between SKAT and MURAT for small window analysis with three different weights. The adjusted SKAT *p*-value is defined as the minimum of two *p*-values obtained by testing LS and FN individually. **A:** identical weights. **B:** Weights based on the Beta (1,25) distribution. **C.** Weights based on Beta (0.5, 0.5).


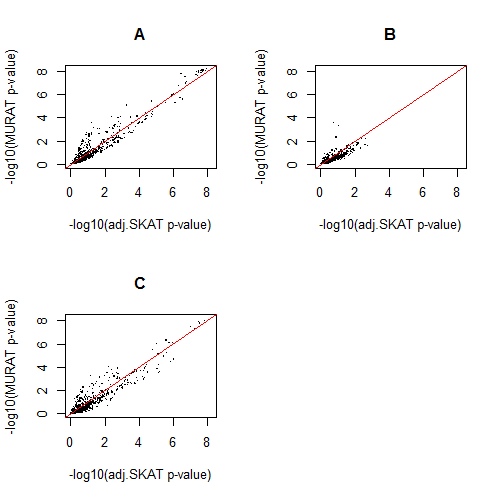


**Supplemental Figure 6:** *p*-value comparisons between SKAT and MURAT in single variant analysis, where the adjusted SKAT *p*-value is defined as the minimum of two *p*-values obtained by testing LS and FN individually.


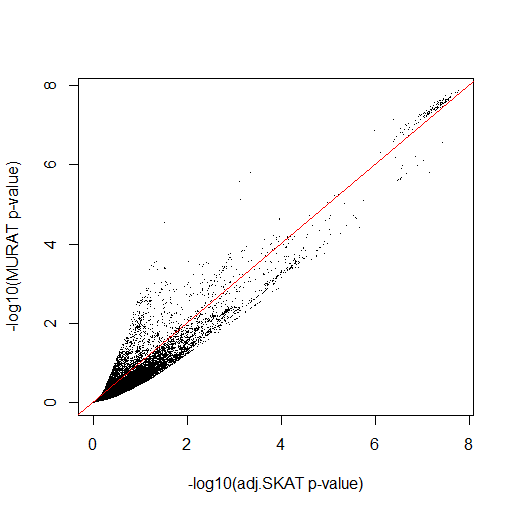


**Supplemental Figure 7:** Box plot of p-values, obtained via MURAT with identical weight for gene pieces analysis, within different groups. Notations G1 to G4 represent group 1 to group 4, , where group 1 has 1360 gene pieces containing less than 100 SNPs; group 2 has 11720 gene pieces containing 100 to 500 SNPs; group 3 has 8670 gene pieces containing 500 to 1000 SNPs; and group 4 has 2583 gene pieces containing more than 1000 SNPs.


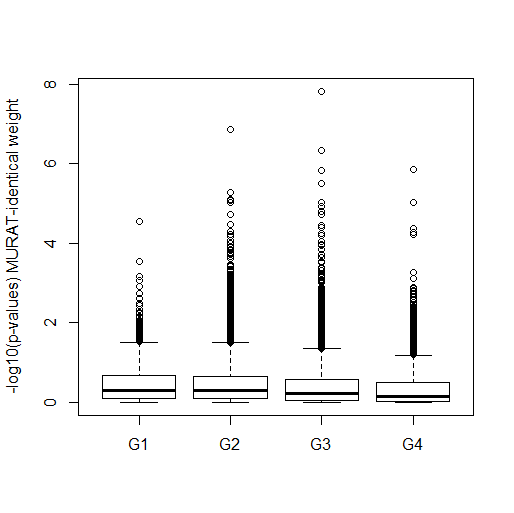


**Supplemental Figure 8:** 95% family-wise confidence level for Tukey’s multiple comparison of mean *p*-values from MURAT analyses of gene pieces grouped by the number of SNPs in the gene pieces. The *p*-values were obtained via MURAT with identical weights. Notations G1 to G4 represent group 1 to group 4, where group 1 has 1360 gene pieces containing less than 100 SNPs; group 2 has 11720 gene pieces containing 100 to 500 SNPs; group 3 has 8670 gene pieces containing 500 to 1000 SNPs; and group 4 has 2583 gene pieces containing more than 1000 SNPs.


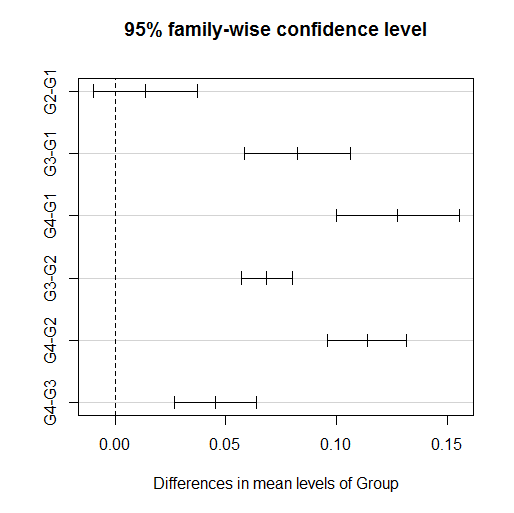


**Supplemental Figure 9:** For 17 selected genes listed in Supplementary Table 3, p-values are compared between models with and without study indicator covariates. MURAT results with identical weights are shown. Similar agreement was seen using other weights and when using SKAT.


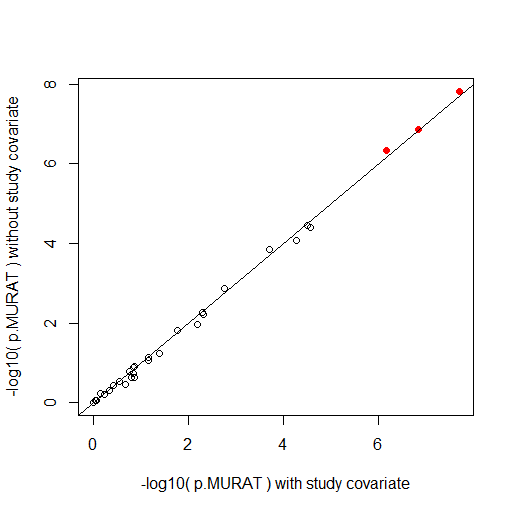

Supplement: Supplementary file 1 — Supplemental Material [file 41598_2017_18385_MOESM1_ESM.docx]
